# A Deep Breath and a Small Sip

**DOI:** 10.1089/pmr.2020.0008

**Published:** 2020-04-30

**Authors:** Wendy Walters

**Affiliations:** UAB Hospital, Birmingham, Alabama, USA.

*Mr. B, a 68-year-old man recently diagnosed with metastatic lung cancer, was admitted to the medical intensive care unit (MICU) with respiratory distress and was subsequently intubated and ventilated. He steadily declined after developing acute respiratory distress syndrome, and his daughter Sheila faced making end-of-life decisions for her father. Mr. B had been living with Sheila and her nine-year-old son Dylan since his wife's death two years prior. The arrangement had been beneficial for all—Sheila had recently divorced and needed support raising her son and Mr. B needed the company and support of his family. Mr. B and Dylan had an especially strong relationship; Mr. B walked Dylan to school daily and served as a father figure to him.*

Sheila struggled with how to tell Dylan about her father's condition and likelihood of death. At the time, I worked as an end-of-life counselor for a large academic hospital in the southeast—I did not work for the palliative care team, but as an independent clinical social worker providing counseling to ICU families facing the death of a loved one. I received a consult from the critical care team to provide support to Sheila about both the medical decisions she was facing and how to talk to her son about dying and death. Sheila's initial instinct was to not allow Dylan to visit even though he begged constantly to see his grandfather. With coaching and a deliberative planned approach to the visit, Sheila decided to allow Dylan to visit Mr. B in the ICU.

I am constantly amazed by the resiliency and wisdom of children. As we planned, Sheila brought Dylan to the door of the ICU and the bedside nurse explained to him, from the doorway, what every tube and machine was doing for Mr. B. By explaining what the machines did, it demystified them and Dylan, like many children of that age, showed enormous curiosity about the technology. Once Dylan was comfortable, he was then willing to come into the room and stand on a stool beside his grandfather's bed. He stunned his mother and the staff by taking Mr. B's hand, telling him “It's OK, Grandpa, we'll be all right.”

I worked with the critical care team as we transitioned this patient and his family into the palliative care service, and eventually to the palliative care unit with a planned compassionate extubation. Dylan was not present when Mr. B died but he arrived shortly thereafter. His mother was acutely grieving and was dreading telling her son that his beloved grandfather had died. We sat in the kitchen of the palliative care unit, and the boy snuggled into his mother's lap. She started to tell him, but dissolved into tears. The boy slipped off her lap, got a cup of water, got back on her lap, handed her the cup, and said, “Mom, what I need from you is a deep breath and a small sip.”

## Abbreviations Used

ICU: intensive care unit
MICU: medical intensive care unit

